# The Thaw Trial

**Published:** 1907-04-20

**Authors:** 


### The Hospital

A Journal of

The Medical Sciences and Hospital Administration.

New Series. No. 3, Vol. I. [No. 1075, Vol. XLII.] SATURDAY, APRIL 20, 1907.

### The Thaw Trial.

The so-called expert witnesses who gave evidence at the Thaw trial confirmed the justice of the scathing definition which Lord Bramwell once gave of expert evidence. In former days this country was not without its scandals, especially in regard to such evidence, tendered chiefly in support of actions against railways after accidents. Happily the evil has greatly diminished in recent times, but there is still something left to desire which might profitably engage the consideration of those who endeavour to exercise some control over the ethics of the profession. It is not possible to give a deliberate judgment, as to the actual mental condition of the prisoner, from the abstracts of evidence which have been cabled to this country. Still the general opinion of mental experts favours the conclusion that no evidence is forthcoming which justifies the belief that the prisoner is insane. One general conclusion may be drawn from the facts which have reached this country, which uphold the view that, had a stronger judge presided over the trial, it might have gone very differently.

In spite of the criticisms which are levelled at our own system of criminal procedure, there can be no doubt that the scandal of the trial would be impossible is an English Court. For many of the discreditable episodes connected with it, American jurisprudence no doubt must not be held responsible, since the wholesome doctrine of "contempt of court" appears to be unknown in New York, or (if it exists) to be treated with derision by newspapers and public alike. But the triumph of technicality over common sense, the tedious system of impanelling a jury, the partisan character of the prosecution, the irrelevancy of the defence, and, above all, the restricted authority of the presiding Judge, have filled English lawyers with amazement. Yet both the American and English systems are derived from a common source—the English Common Law; and the causes of their divergence are to be looked for in differences not so much of principles as of method. It is to the position and authority enjoyed by an English judge, backed up by a sane and sober public opinion, that we owe the restriction put upon technicalities which are still in theory possible in our own Courts, and the keeping of evidence, even on the prisoner's behalf, within reasonable bounds. An English Judge has always the support of the strong professional feeling of the Bar.

The issue in the Thaw case was clear; yet it was six months before the prisoner was called on to plead, and nearly another four before the jury finally disagreed. Justice in such circumstances loses not only its dignity but its moral effect, to say nothing of the encouragement afforded to wealthy criminals who can command the services of ingenious representatives. It is not for us to convict Thaw or to acquit him, though it would not be difficult to guess the verdict of an Old Bailey jury. The contrast of the trial of the Whiteley murderer is still fresh in the public mind, and it will be long before even Home Secretaries are able to destroy the sure instincts of English jurymen or the dignity and common sense of the English Bench.

The moral issues involved in this case have a very serious bearing upon the government of all civilised countries. The people in most civilised nations, in regard to certain vices, are in a state of transition, but the time has arrived when, in the interests of public morality, and for the protection of the innocent, the law should be strengthened, so as to bring into its meshes every wealthy voluptuary who uses his wealth, and the power which it gives him to destroy innocent children. All convicted of such crimes should be condemned to the madhouse or the prison for the rest of their existence. It would be unreasonable for the English people to plume themselves upon their superior morality, seeing that we have reason to believe that the Madison Tower chamber may have its counterpart in this country, and that most, if not all, of its details were modelled upon those of similar places to be found in most great cities. We have always felt that the abiding scandal of many streets in our large cities should be faced and ended by setting aside one area in each community to which houses of ill fame should be restricted, and where every occupied dwelling should be continuously inspected by the authorities, under a system which should render most of the existing evils impossible of continuance. Under such a system our principal streets in the evenings would be freed from the present abuses which make them impossible to be used in comfort by respectable people at certain hours. Then, too, all persons who voluntarily entered a house of ill fame would have to incur the risk of publicity, because to do so they must enter and leave the prescribed area. The present English method of maintaining that in this country the vice of cities is relatively unimportant must be held responsible for the sacrifice of many young people of both sexes, who might otherwise be saved from destruction. The action of Mr. W. T. Stead some years ago led to useful legislation, which has had some effect in controlling vice. But we English people need to-day a wholesome public conscience, which should compel the municipalities to cleanse our streets from their existing impurities, by localising vice and bringing the whole of its machinery, despite the wealth upon which it may rest, within the iron grip of the law.

